# Designing transformative sustainability education: a mixed methods study exploring the values and perspectives of medical students

**DOI:** 10.1186/s12909-026-09960-8

**Published:** 2026-07-31

**Authors:** Tim Smith, Fiona Muir, Elizabeth Lakin, Shona Livingstone, Chantelle Rizan

**Affiliations:** 1 Department of Surgery and Critical Care, University of Otago, Christchurch, New Zealand; 2 University of Dundee School of Medicine, Dundee, UK; 3 National University of Singapore, Singapore, Singapore; 4 University of Dundee School of Humanities, Social Science and Law, Dundee, UK

**Keywords:** Education for sustainable healthcare, Transformative learning, Environmental values, Curriculum co-design

## Abstract

**Introduction:**

Education for Sustainable Healthcare (ESH) is a novel area of medical education and high-quality evidence to guide effective learning approaches is yet to be established. This study investigates the design of ESH in undergraduate medicine through the lenses of transformative learning, environmental value position and student-centred design.

**Methods:**

A mixed methods approach was used. A questionnaire assessed the environmental value position and attitudes towards ESH of undergraduate medical students at the University of Dundee. Semi-structured interviews with a sub-group of these students further evaluated environmental perspectives and explored preferences for learning approaches for ESH.

**Results:**

The questionnaire data indicated that the students had high levels of environmental concern, arising from an ecocentric value position. Ecocentrism correlated with support for the integration of ESH and sustainability in medicine. The qualitative findings from 10 interviews confirmed high levels of environmental concern and support for ESH but suggested a more complex underlying value basis. There was a disconnection between students’ environmental values and their engagement with sustainable medical practice, arising from conflict between their sense of medical professionalism and aspects of sustainable medical practice that were perceived to be at odds with providing optimal patient care. Regarding learning approaches, students highlighted the need to prioritise examined content, and reported a preference for small group, interactive learning, delivered by engaged tutors in an authentic and relevant way.

**Discussion:**

It is important that the integration of ESH involves summative assessment; without this, students are unlikely to engage with the topic. ESH learning approaches should be designed in accordance with student preferences if they are to maximise impact: interactive, student-centred, experiential learning approaches are preferred. ESH should bridge the gap between students’ environmental attitudes and capacities for sustainable behaviour by empowering them to act sustainably through role modelling, skills acquisition, project work and critical reflection.

**Supplementary Information:**

The online version contains supplementary material available at https://doi.org/10.1186/s12909-026-09960-8.

## Background

Recognition of the health effects of environmental degradation has resulted in calls for Education for Sustainable Healthcare (ESH) to be integrated into undergraduate medical curricula [1–3], the aim of which is to produce a healthcare workforce capable of supporting sustainable models of patient care and engaging in environmental advocacy [4–6]. This represents a fundamental shift in health professionals’ duties and responsibilities, including taking on leadership roles addressing local and global environmental challenges [2]. Evidence to inform the design of ESH approaches that prepare students to meet these sustainability duties and result in positive changes in environmental behaviour is limited; empirical studies reporting ESH interventions do not appear to assess outcomes which reflect these transformative changes in health professionals’ roles [7, 8].

Recognising the need for transformative change in addressing sustainability challenges, educators from other disciplines have adopted alternative learning approaches to sustainability teaching and assessment [9–11], underpinned by Transformative Learning Theory. Transformative Learning Theory, originally developed by Mezirow [12], represents a higher order form of learning [13] which leads to a fundamental shift in an individual’s worldview and epistemic beliefs [12, 14] and results in a sense of agency to effect change through positive action [15].

The ESH literature makes limited reference to this approach [16–18], with some educators advocating for a reframing of healthcare systems and wider society via transformative approaches to learning; however, there is a gap between these calls for transformative learning and the reality of ESH delivery. Recent systematic reviews exploring the strategies used in sustainable healthcare and planetary health education [7, 8] suggest that this learning theory does not inform either the design or the impact assessment of the majority of ESH interventions, and so it remains unclear how to implement transformative learning in this novel area of medical education.

Applying transformative learning in sustainability education requires an understanding of students’ environmental worldviews [19], since it involves a fundamental shift in their pre-existing values and beliefs [12]. Although there are a number of studies reporting medical students’ attitudes towards topics such as climate change and sustainable healthcare [20–22], there is an absence of research assessing their underlying environmental value position (EVP), either quantitively or qualitatively. In addition, no attempts have been made to relate medical students’ EVP to their willingness to engage in ecologically sustainable clinical practice (ESCP). The rationale for exploring this relationship is that any positive or negative associations found may be helpful to inform the way in which ESH teaching is delivered in terms of content, framing and pedagogical approaches [19].

Individuals may be said to hold a value position that lies on a spectrum of environmentalism, ranging from ecocentric to anthropocentric [23] p.67. An ecocentric position holds that nature has intrinsic value, and recognises the ecosystem level as the central object of moral concern [24]. Anthropocentrism on the other hand, holds that humans are of primary importance and separate from nature, and considers nature to possess instrumental value, providing us with tangible services such as food, fuel, timber and fibre, and, for that reason, some environmental problems need to be addressed. It has been suggested that the ecocentric commitment to the intrinsic value of nature translates into a greater likelihood of taking positive environmental action [25], with proponents of ecocentrism arguing that an anthropocentric (human-centred) value system is at the root of current ecological crises. Critics of ecocentrism however, contend that an anthropocentric worldview can be a powerful motivational force for environmental protection, if it is understood that human health and survival is entirely dependent on intact ecosystems and biodiversity [26].

Furthermore, it has been suggested that student involvement in curriculum co-design has the potential to not only enhance their engagement [27], but may also result in transformative changes in the way they think and behave [28]. Whilst there are examples of stakeholder involvement in ESH research [29, 30], in-depth, qualitative exploration of medical student preferences for ESH delivery appears to be lacking. The aim of this research is to address these two gaps in the peer reviewed literature, exploring the design of transformative learning approaches for ESH in undergraduate medicine from the perspectives of environmental value position and student-centred design.

## Methods

### Study design

#### Sequential mixed methods strategy of enquiry

Two methods of data collection (an online questionnaire and semi-structured interviews) were used to address the research questions, representing a sequential mixed methods strategy of enquiry.

### Quantitative methods

#### Aims and objectives

The aim of the quantitative study was to assess the EVP of the medical students at the University of Dundee and relate this to their willingness to engage in sustainable medical behaviour, expressed here as Ecologically Sustainable Clinical Practice (ESCP), to help inform the design of ESH in undergraduate medicine. Sustainable medical behaviour refers to a range of intended behaviours such as the adoption of low carbon clinical interventions, practicing realistic medicine, rational use of prescribing and investigations, green and social prescribing, waste reduction and environmental advocacy.

#### Sampling, participants and distribution

A questionnaire was emailed to all undergraduate students enrolled in the MBChB course at the University of Dundee School of Medicine. This included students in years one to five, those undertaking an intercalated BMSc qualification, and those in the Scottish Graduate Entry Medicine (ScotGEM) MBChB programme, a total of 1243 students. The questionnaire is shown in the Supplementary Data File.

#### Data collection instrument

The Ecocentric and Anthropocentric Attitudes Towards the Environment (EAATE) assessment tool [25] was used to investigate EVP. The EAATE tool consists of 33 statements related to the natural environment. Respondents were asked to rate their agreement or disagreement to these, using a five-point Likert Scale. Additional questions were created to assess students’ attitudes towards ESH and ESCP. The questionnaire generated four sub-scale scores: ecocentrism, anthropocentrism, apathy and ESCP. EAATE and ESCP items are shown in the Supplementary Data File.

#### ESCP subscale

A succinct, purpose-built scale was developed to assess willingness to engage in sustainable clinical behaviour, as no validated instrument exists to quantify this concept. Items were designed to capture key dimensions of ESCP, informed by relevant ESH literature, for example: [2, 3] and by the authors’ experience of teaching ESH to undergraduate medical students and involvement with instituting sustainable quality improvement measures into clinical practice. The questions were reviewed by the research team for face and content validity. Formal pilot testing and construct validation were not undertaken, reflecting the exploratory nature of this study. Statistical checks for reliability were conducted (Cronbach’s Alpha). The scale was developed to support the design of ESH within the context of this research, rather than establish a definitive measurement instrument, thus, the results derived from this scale are interpreted cautiously and are intended to inform and integrate with the qualitative findings.

#### Inferential statistics

##### Non-parametric tests

The subscale data was assessed for normality using graphical methods, the Shapiro-Wilk Test, and tests of skewness and kurtosis. Subscale scores were compared between gender groups, ethnic groups, and for year of study. Due to the non-normality of the subscale data, non-parametric tests were used to compare ESCP scores and assess the statistical significance of any observed differences between demographic groups.

##### Correlation analysis

Spearman’s Rank Order Correlation was used to investigate monotonic associations (linear and non-linear) between the continuous variables: age and subscale scores (ecocentrism, anthropocentrism, apathy and ESCP).

##### Regression analysis

Regression analysis was undertaken, to further understand the relationships between the variables, adjust for variables, and generate an explanatory model involving the dependent variable, ESCP, and the independent variables, which included the other subscale scores (ecocentrism, anthropocentrism and apathy) and the demographic variables. Given the skewed nature of the data, robust regression was used to fit these models, being an alternative to Ordinary Least Squares linear regression that is less sensitive to outliers or observations with undue influence.

### Qualitative methods

#### Aims and objectives

The objectives of the qualitative study were to triangulate the environmental value position data obtained from the questionnaire, comparing this to students’ attitudes and opinions expressed in the interviews. In addition, taking a user-centred approach, the study sought to explore the preferred learning methods of medical students, to generate ideas for transformative approaches to ESH.

#### Sampling and participants

In the recruitment email for the questionnaire in the quantitative study, students could opt-in to take part in the subsequent semi-structured interviews. Convenience purposive sampling was used, due to the limited number of respondents who opted in to participation in the interviews, and the numbers within that subgroup who subsequently responded to email invitations to attend an interview.

#### Data collection

##### Semi-structured interviews

Semi-structured interviews were chosen as the data collection technique as it is well-suited to exploring situations in which there is little prior data and minimal assumptions about what the participants will share, and allows a deep understanding of the issues to be obtained [31]. The interview guide is shown in the Supplementary Data File. The first part of the interview process focused on the student’s attitudes towards general environmental issues, their thoughts about the integration of ESH in undergraduate medicine, and the role of doctors in protecting the natural environment. The second part aimed to understand student preferences for learning approaches in general, and to explore their ideas about how planetary health and sustainable healthcare might be taught in such a way as to result in positive behavioural changes.

Students were given the option to hold the interviews online, via Microsoft Teams, or in-person. For those interviews conducted in person, Yuja Software was used for recording and transcription. For the Teams interviews, the in-built recording and transcription features were used. The automatically generated transcripts were reviewed together with the recordings, to ensure an accurate representation of the interviews.

Data collection, transcription and analysis were performed concurrently. Interviews were undertaken until data saturation was reached, at the point where no substantively new ideas or insights were being generated by subsequent interviews, similar comments were being repeated, and no new themes were emerging. A further two interviews were undertaken to improve confidence that data saturation had been reached.

#### Data analysis

Thematic analysis was selected as the analytical approach. Braun and Clark’s [32] six-stage framework was chosen to guide the process. The process was iterative, involving repeated interrogations of the data, generating codes and themes, looking for connections and making refinements. Interview transcripts were uploaded to NVIVO software, re-reviewed, annotations made, key text highlighted, and initial codes created. Codes were a mixture of a priori main codes (pre-established based on the research questions and literature) and emergent sub-codes.

The next stage involved analysis across the interviews, reviewing the initial codes and related text, looking for connections and relationships between codes, and merging and refining the codes. From here, the data and codes were reviewed once again, searching for initial themes. Themes were elements that were obviously repeated across multiple interviews, or of key importance to the research questions. Initial themes were reviewed, refined and organised, via re-reading of the data, and using mind mapping as a technique for visualising the codes and themes, and looking for relationships. The thematic analysis process was both deductive and inductive; the overarching themes or concepts were based on the research questions; however, major themes and sub-themes were allowed to emerge from the data. Many of these inductive themes were chosen based on their prevalence within the data, however others were included as they were judged to provide a more complete picture of the main concept.

Member checking was not used, as the themes that emerged represented the lead researcher’s interpretation of the data rather than a simple reflection of the participants’ responses. However, emerging themes were discussed with the wider research team, and rigour was further enhanced through peer debriefing, reflexive journalling and maintenance of a clear audit trail.

### Researcher positionality

The lead researcher held a teaching and leadership role at the University of Dundee School of Medicine at the time of conducting the questionnaire and interviews. This power imbalance had the potential to cause students to feel obliged to participate or answer questions in a particular way (such as over-reporting of environmental concerns or reluctance to express scepticism). These risks were mitigated by outlining the non-obligatory nature of participating in either aspect of the research (via the email invitations and participant information sheets), the ability to remain anonymous if participating in the questionnaire, stressing the collaborative and participatory nature of the interview process, and by ongoing reflexivity throughout the research process. The interview process was designed to foster an environment of openness, trust and participation. Prior to the commencement of each interview the collaborative nature of the research was reiterated: it was stressed that there were no right or wrong answers, rather that the aim was to gather a diverse range of students’ attitudes and opinions in order to design effective ESH teaching sessions.

## Results

### Questionnaire results

#### Scale reliabilities

Cronbach’s Alpha was calculated for the individual subscales to assess how closely related the subscale items were as a group. Cronbach’s Alpha was greater than 0.7 for the three EAATE subscales and was determined to be 0.74 for the newly created ESCP subscale, thereby indicating an acceptable level of reliability for all subscales [33]. A table of results is shown in the Supplementary Data File.

#### Student demographics

One hundred and eight out of 1,243 undergraduate medical students responded, giving a response rate of 8.7%. A summary of respondent demographics is shown in Table 1.

**Table 1:** Demographics

Demographics		Number of participants (%)
Year of study	Year 1	27 (25)
	Year 2	20 (18.5)
	Year 3	15 (13.9)
	BMSc intercalating	12 (11.1)
	Year 4	8 (7.4)
	Year 5	15 (13.9)
	ScotGEM 3	4 (3.7)
	ScotGEM 4	7 (6.5)
Gender	Female	73 (67.6)
	Male	31 (28.7)
	Other	2 (1.9)
	Prefer not to say	2 (1.9)
Ethnicity	White/ Caucasian	86 (79.6)
	Asian	14 (13)
	BACBB	3 (2.8)
	Arab	1 (0.9)
	Mixed/ multiple	3 (2.8)
	Other ethnic group	1 (0.9)

*BMSc* Bachelor of Medical Sciences, *ScotGEM*  Scottish Graduate Entry Medicine, *BACBB* Black/ African/ Caribbean/ Black British

#### Tests of normality

The distribution of the data for ESCP in percentage units showed strong evidence of departure from normality (Shapiro-Wilk normality test: *p* < 0.001), which was not removed by log transformation.

#### Descriptive statistics

Descriptive statistics of the results of the subscale scores are presented in Table 2. The mean Ecocentrism score was 85.6%, the mean Anthropocentrism score was 54.6%, the mean Apathy score was 29.1% and the mean ESCP score was 84.4%. The dependent variable, ESCP (expressed as percentage scores), split by gender, ethnic group and year of study is shown in Table 3. The EAATE subscale scores (expressed as percentages) split by demographic variables are shown in the Supplementary Data File.

**Table 2:** Subscale percentage scores

Subscale	*N*	Mean % (SD)	Median % (IQR)	Minimum %	Maximum %
Ecocentrism	108	85.57 (8.52)	86.67 (11.67)	51.67	100.00
Anthropocentrism	108	54.63 (9.78)	55.00 (11.25)	30.00	81.67
Apathy	108	29.07 (8.84)	26.67 (11.11)	20.00	57.78
ESCP	108	84.44 (11.22)	83.33 (16.67)	53.33	100.00

*SD*  Standard Deviation, *IQR* Interquartile Range

**Table 3:** ESCP percentage scores split by demographic variables:

Demographic	Subgroup	*N*	ESCP %	
			Mean (SD)	Median (IQR)
Gender	Female	73	84.47 (10.99)	83.33 (16.67)
	Male	31	83.33 (11.71)	83.33 (16.67)
	Other	2	100 (0)	100 (0)
	PNTS	2	85.00 (11.79)	85.00 (-)
Ethnicity	WC	86	85.74 (10.81)	86.67 (16.67)
	Asian	14	78.81 (11.95)	76.67 (15.00)
	BACBB	3	78.89 (7.70)	83.33 (-)
	Arab	1	60.00 (-)	60.00 (-)
	MM	3	88.89 (10.18)	86.67 (-)
	Other	1	80.00 (-)	80.00 (-)
Year of study	1	27	79.50 (12.25)	80.00 (16.67)
	2	20	87.33 (10.90)	86.67 (19.17)
	3	15	84.44 (10.89)	83.33 (20.00)
	BMSc	12	90.28 (10.96)	93.33 (16.67)
	4	8	80.00 (9.09)	80.00 (15.00)
	5	15	85.33 (9.41)	83.33 (16.67)
	ScotGEM3	4	86.67 (15.15)	90.00 (28.33)
	ScotGEM4	7	87.14 (7.56)	86.67 (10.00)

*PNTS* prefer not to say, *WC* White/ Caucasian, *BACBB* Black/ African/ Caribbean/ Black British, *MM* Mixed/ Multiple, *BMSc* Bachelor of Medical Sciences, *ScotGEM* Scottish Graduate Entry Medicine

#### Likert data

The EAATE scale consists of 33 statements, used in conjunction with a five-point Likert scale to calculate scores for three subscales: Ecocentrism, Anthropocentrism and Apathy. An additional six statements were added to create an ESCP (Ecologically Sustainable Clinical Practice) scale for assessing students’ attitudes towards engaging in sustainable medical behaviour.

##### Ecocentrism subscale

**Fig. 1:**
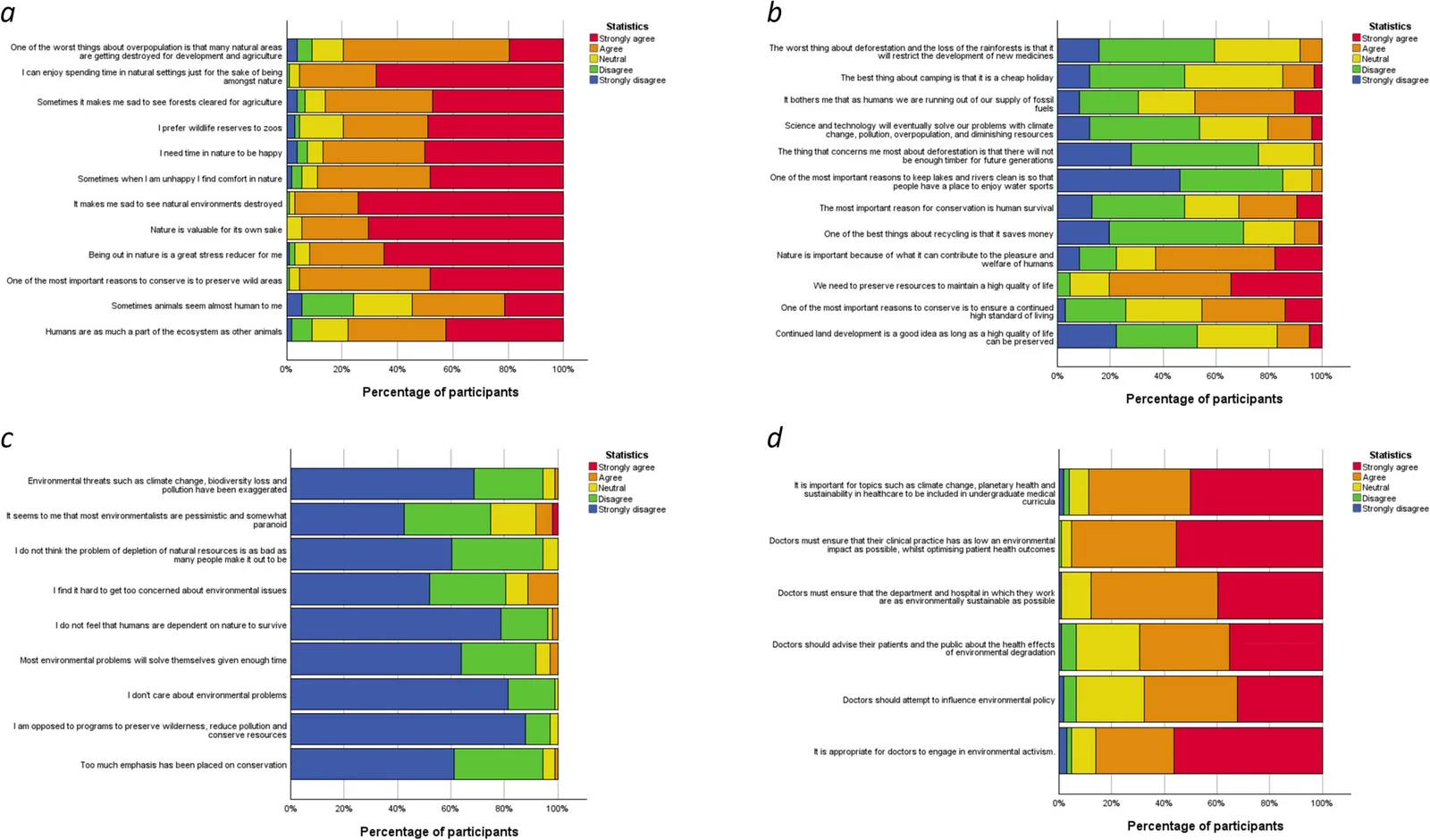
** a** Ecocentrism subscale items.** b** Anthropocentrism subscale items.** c** Apathy subscale items.** d** ESCP subscale items

The Ecocentrism Subscale consisted of 12 items, designed to assess the degree to which respondents subscribed to an ecocentric worldview. The results of the individual items are presented as a Likert plot in Fig. 1a with the responses expressed as percentage of participants. The results show agreement, or strong agreement with an ecocentric environmental value position, reflecting the high mean ecocentrism score (85.6%) found in this sample population.

##### Anthropocentrism subscale

The Anthropocentrism Subscale also consisted of twelve items, assessing the degree to which students hold a human-centred, instrumental view of nature, in which environmental protection is deemed important for the benefits this would confer on humans. The Likert plots in Fig. 1b show a tendency towards neutrality or disagreement with an anthropocentric environmental value position, which is reflected in the more modest mean anthropocentrism score (54.6%), in comparison with the mean ecocentrism score (85.6%).

##### Apathy subscale

The Apathy Subscale consists of nine items and measures the degree to which students are disengaged or ambivalent about environmental issues. The Likert plots in Fig. 1c show a strong disagreement with environmental apathy, reflecting the low mean score (29.1%) for this subscale overall.

##### ESCP subscale

The ESCP (Ecologically Sustainable Clinical Practice) Scale assessed students’ attitudes towards the introduction of sustainability teaching in medicine, and their willingness to engage in various types of sustainable medical behaviour, including advocacy and environmental activism. ESCP score is the dependent variable in the context of the inferential statistical analysis. The Likert plots in Fig. 1d show agreement or strong agreement with sustainability in medicine, which is reflected in the high mean ESCP subscale score (84.4%).

#### Inferential statistics

##### Non-parametric tests

Non-parametric tests were used to compare ESCP percentage scores, to assess for statistically significant differences between demographic groups. A statistically significant ethnicity difference in the median ESCP percentage score was observed. The median score was 10% units lower in Asian compared to White/ Caucasian students, and this difference was statistically significant at the 5% level (Mann-Whitney U = 296, *p* = 0.029). The Asian student subgroup comprised only fourteen participants, substantially limiting the statistical power of this comparison and no generalisable conclusions can be drawn. The median ESCP percentage score was identical for males and females. There was no statistically significant difference in median ESCP percentage score between gender groups or by year of study (grouped into Years 1–3, BMSc and Years 4–5/ ScotGEM). The results are shown in the Supplementary Data File.

##### Correlation analysis

Spearman’s rank correlations were calculated between the continuous variables (subscale scores and age), and these are presented in Table 4. It can be seen from Table 3 that there was strong statistical evidence for a positive association of moderate strength (*r* = 0.48, *p* < 0.001) between ecocentrism score and ECSP score, and evidence of a weak positive association between age and ESCP score (*r* = 0.22, *p* = 0.023). In addition, there was statistical evidence for a weak negative association (*r* = 0.20, *p* = 0.042) between anthropocentrism and ESCP scores, but a weak positive association (*r* = 0.29, *p* = 0.03) between anthropocentrism and apathy scores. Furthermore, a statistically significant negative association of moderate strength (*r* = 0.54, *p* < 0.001) was found between apathy and ESCP score.

**Table 4:** Spearman’s Rank Correlation Coefficients for subscale scores and age

		Ecocentrism	Anthro-pocentrism	Apathy	ESCP	Age
Ecocentrism	Correlation Coefficient (*r*)	1.000	− 0.093	− 0.498^**^	0.482^**^	0.128
	p-value (2-tailed)	.	0.341	< 0.001	< 0.001	0.185
Anthro-pocentrism	Correlation Coefficient (r)	− 0.093	1.000	0.285^**^	− 0.196^*^	− 0.053
	p-value (2-tailed)	0.341	.	0.003	0.042	0.586
Apathy	Correlation Coefficient (r)	− 0.498^**^	0.285^**^	1.000	− 0.540^**^	− 0.063
	p-value (2-tailed)	< 0.001	0.003	.	< 0.001	0.519
ESCP	Correlation Coefficient (r)	0.482^**^	− 0.196^*^	− 0.540^**^	1.000	0.219^*^
	p-value (2-tailed)	< 0.001	0.042	< 0.001	.	0.023
Age	Correlation Coefficient (r)	0.128	− 0.053	− 0.063	0.219^*^	1.000
	p-value (2-tailed)	0.185	0.586	0.519	0.023	.

*ESCP* Ecologically Sustainable Clinical Practice

**. Correlation is significant at the 0.01 level (2-tailed)

*. Correlation is significant at the 0.05 level (2-tailed)

##### Regression analysis

Univariable and age adjusted analyses allowed certain variables (gender, ethnicity and year of study) to be excluded from the subsequent multiple regression analysis, as there was little evidence of an effect on ESCP. Summarising the final multiple regression model, shown in Table 5, there is a positive association of ESCP with ecocentrism score: a 1 unit increase in ecocentrism score is associated with a 0.41 increase in ESCP score. There is a negative association of ESCP with apathy score: a 1 unit increase in apathy score is associated with a 0.48 decrease in ESCP score. There is weak evidence of a positive association of ESCP with age: a 1 unit increase in age results in a 0.51 increase in ESCP score.

**Table 5:** Final robust regression model

	Coefficient	Standard error	Z value	*P*-value	F-statistic	F-test *p* value
Intercept	63.35	13.29	4.77	<0.0001	-	-
(Age – 21 ^+^)	0.51	0.29	1.72	0.086	2.98	0.087
Apathy %	-0.48	0.12	-3.98	0.0001***	15.08	0.0002
Ecocentrism %	0.41	0.13	3.19	0.001**	10.35	0.002

^+^Age in years centred at the median of 21. Significance: * < 0.05; **<0.01; ***<0.001

### Interview results

#### Student demographics

The demographics of the 10 student participants are outlined in the Supplementary Data File.

#### Themes

Major themes and sub-themes are described below, and a summary table is presented in the Supplementary Data File. Quotations are used within the descriptions to help illustrate the themes that emerged and enhance reader understanding, and to give the students a voice in the research. Students’ ecocentrism, anthropocentrism, apathy and ESCP sub-scale scores are shown in brackets following quotations, to facilitate a comparison between the quantitative assessment of their environmental value position, and the attitudes and values they expressed in the semi-structured interviews.

##### Human-centredness

When discussing environmental value position, 7 out of the 10 participants made comments that suggested a tendency towards a more anthropocentric worldview. Their statements reflected a desire to protect the natural environment, but for human-centred reasons, rather than for its intrinsic value: *“I think I’m right in the middle… where it’s from a human-centred perspective*,* because that’s the only perspective you actually have.”* Participant 10 (Eco: 44, Anthro: 39, Apathy: 18, ESCP: 22). Of the participants expressing this viewpoint, some were consistent with their EAATE scores, whereas others were not.

##### Emotional turmoil

The students expressed a variety of negative emotional responses expressed in relation to current ecological crises. The most predominant emotions were frustration, guilt and anxiety, with some students expressing a mixture of these. Frustration refers to the students’ perception that they were making changes and sacrifices to reduce their individual ecological footprint, however this was not accompanied by action being taken by those in influential leadership positions, such as governments and multinational corporations: *“I can never use a plastic bottle ever again and I can never buy a piece of clothing ever again*,* and just live completely waste free*,* and it still won’t really matter that much compared to all the private jet flights.”* Participant 6 (Eco: 49, Anthro: 32: Apathy: 13, ESCP: 24).

Guilt reflects the conflict surrounding individual behaviour (e.g. flights, dietary choices), and whether they could be doing more to help, or consuming less, to reduce environmental impact: *“Myself and my girlfriend went to Thailand and Vietnam… on one side I’m thinking of the carbon footprint of all of that traveling and tourism… but at the same time I don’t want to restrict myself from having these experiences.”* Participant 4 (Eco: 57, Anthro: 26, Apathy:10, ESCP: 29).

Anxiety about the current and future effects of environmental degradation, especially climate change, was expressed: *“I avoided flying abroad for a while*,* and I ate a vegetarian diet because I understood that was a good way to minimize my impact… I had quite a bit of what you might call climate anxiety”.* Participant 7 (Eco: 47 Anthro: 37 Apathy: 21 ESCP: 25).

##### Ecological health consciousness and support for ESH

All the students expressed concern for the environment, including the impact of healthcare. Nine of the 10 students expressed support for ESH. Many of the students rationalised their support for ESH based on the links between the environment and human health: *“How can you care about health but not the environment when the environment causes such an impact on health.”* Participant 3 (Eco: 57, Anthro: 41, Apathy: 11, ESCP: 25).

##### Conflict with respect to sustainable clinical practice

This theme represents the sense of conflict experienced by the students, arising from their desire to prevent further environmental degradation caused by the negative impacts of the healthcare industry, which was perceived to be at odds with their professional responsibility to put patient care first: *“There’s only so much you can do as a doctor because safety needs to come first… if you care too much about the planet*,* and you stop using gloves and aprons then you’re putting people’s safety at risk*,* but if you care too much about people*,* there might not be a planet.”* Participant 2 (Eco: 54, Anthro: 37, Apathy: 12, ESCP: 24). The EAATE scores indicated that students with high ecocentrism scores also expressed the view that patient care supersedes environmental protection.

Waste generation appeared to be a significant contributor to this sense of conflict. The data included repeated references to the large amount of waste (particularly single-use plastic items) students had observed on their clinical attachments. There was an appreciation of the rationale behind this, in terms of infection control, but also a sense of frustration: *“Most equipment is plastic… the packets are plastic… the tubes are plastic. It’s just hard to change that because there’s no alternative”* Participant 10 (Eco: 44, Anthro: 39, Apathy: 18, ESCP: 22).

##### Support for environmental advocacy

Six students suggested engaging in different forms of environmental advocacy. Some suggested the use of patient consultations as an opportunity to discuss to health behaviour of relevance to the environment (such as diet and active travel): *“I would definitely try and counsel patients… even though it’s not part of our teaching in communication skills to counsel patients on planetary health*,* I can implement it anyway.”* Participant 5 (Eco: 51, Anthro: 26, Apathy: 9, ESCP:30). Students were asked whether environmental advocacy extended to activism and civil disobedience. Five of the participants supported doctors taking part in activities such as climate change demonstrations and non-violent social protests. However, this was tempered with conflicting feelings about the role of doctors in society and concerns about professionalism.

##### Preference for active learning

Students were asked about their preferences for learning approaches, and for their ideas for integrating ESH topics into the curriculum. All the students expressed a positive opinion about the value of active learning approaches, in particular small group sessions: *“Put it into smaller sessions… discussing it with your peers… tie it into whatever is going on that week”* Participant 1 (Eco: 53, Anthro: 48, Apathy: 11 ESCP: 25). Small group work included assignments and discussions, debates and communication skills sessions: *“… you could practice how to advocate planetary health … small group teaching*,* leading to an OSCE”* Participant 5 (Eco: 51, Anthro: 26, Apathy: 9, ESCP:30). Students also described other types of active learning activities such as assignments and project-work, ideally associated with incentives such as rewards or prizes: *“others… are unlikely to engage in something that’s optional and there’s no obvious reward… to cover that group of people you would introduce an incentive… because medical students like a challenge”* Participant 4 (Eco: 57, Anthro: 26, Apathy:10, ESCP: 29).

##### The necessity of relevance

All students expressed that ESH needs to be delivered in a way that is relevant to medical students and doctors. Clinical relevance was seen as important; for example, providing teaching about the negative health impacts of different types of environmental degradation, such as air pollution: *“Small group work*,* that’s presented to us from a clinical point of view*,* because I think I respond the best to that”* Participant 6 (Eco: 49, Anthro: 32: Apathy: 13, ESCP: 24). Providing clarity about the role of doctors in contributing to sustainable healthcare, and practical teaching to reduce the environmental impact of their daily routine was valued. For example, teaching about the rational use of medical investigations, such as blood tests, from the perspective of reducing waste and greenhouse gas emissions.

Many students commented on the volume and intensity of the undergraduate medical curriculum, and the need to prioritise examined content, the implication being that sustainable healthcare topics need to be summatively assessed, otherwise they will be ignored: *“Environmental health is not a topic that most people find is a priority in medical school… if you could add it to the exam curriculum*,* everybody’s going to do it 100%… everything needs to be related to exams. As a student*,* it’s just too high of a priority to ignore.”* Participant 10 (Eco: 44, Anthro: 39, Apathy: 18, ESCP: 22). Some students mentioned prizes that enhanced their portfolios (a structured collection of evidence supporting their learning and professional development) or curriculum vitae as a way of incentivising students to engage with ESH.

##### The importance of authenticity

It was important to the students that ESH teaching was delivered in an authentic way. Tutor engagement and enthusiasm for a topic was perceived as essential for determining the effectiveness of a teaching session: *“We can tell definitely if somebody is just reading a script that they were given by the medical school*,* or if somebody actually cares about it… and can explain to us how planetary health matters for the communities we’re going to work with”* Participant 6 (Eco: 49, Anthro: 32: Apathy: 13, ESCP: 24). Authenticity also refers to the importance of role-modelling of sustainable healthcare activities, which might include learning about examples of sustainability initiatives and metrics in their own university or hospital.

## Discussion

This mixed methods research explored transformative ESH through the lenses of EVP and student-centred design. A summary of the findings, integration and recommendations is shown in the Supplementary Data File. Overall, the quantitative and qualitative findings highlight several elements that should be taken into consideration when integrating ESH into undergraduate medical curricula. Firstly, the likelihood of high pre-existing levels of environmental concern in medical students; secondly, the barriers that underlie the gap between their motivation to act and their perceived ability; and thirdly, their preferences for ESH design and delivery.

### Environmental value position

The results of the questionnaire demonstrate high levels of environmental concern. This is consistent with the findings of other studies of medical student attitudes towards environmental issues [21, 30, 34, 35]; however, whilst other studies of sustainability in healthcare settings have attempted to assess general levels of environmental concern, this research is unique in its exploration of the underlying value systems that motivate pro-environmental medical behaviour. The quantitative findings showed high ecocentrism, moderate anthropocentrism, and low apathy scores, reflecting strong support for environmental protection generally. The questionnaire also assessed attitudes towards ESH and willingness to engage in pro-environmental medical behaviour, referred to here as ecologically sustainable clinical practice (ESCP), and found that the students held high ESCP scores, reflecting strong support for sustainability within medical education and practice. The finding of an ethnic difference in ESCP (10% units lower in Asian compared to White/ Caucasian students) should be interpreted with considerable caution; the Asian student subgroup was very small (*n* = 14), which substantially limits the statistical power of this comparison. The subgroup analyses were exploratory in nature and conducted to highlight potential patterns that may warrant further investigation.

Correlation analysis and regression modelling of the EVP data demonstrated that ecocentrism was significantly positively associated with ESCP. On the other hand, anthropocentrism was significantly negatively correlated with ESCP and showed a positive correlation with apathy. Although there are many factors that influence environmental behaviour [36], these results suggest that students holding an ecocentric (as opposed to an anthropocentric) worldview are more likely to be receptive to ESH in the curriculum and more willing to engage in sustainable clinical practice.

The response rate (8.7%) and potential sampling bias in the quantitative study limits the generalisability of the EVP findings. However, this was an exploratory mixed methods study involving ESH design, with greater weighting placed on developing a richer, more in-depth understanding of EVP and how it might affect learning approaches for ESH. Consequently, the qualitative findings corroborate the high levels of environmental concern and support for ESH demonstrated by the quantitative study, but suggest a more complex underlying value basis, and less deeply held ecocentric worldviews, particularly in the context of medicine. Interpreting these findings, it might be speculated that a more human-centred worldview (aligning more with anthropocentrism) is to be expected amongst medical students, given the nature of their future profession, which may or may not be accompanied by a more general concern for the intrinsic value of nature. Dietz [37] noted that in some studies evaluating ecocentrism and anthropocentrism, respondents do not always demonstrate this distinction in value systems, with survey items assessing these different concepts loading on the same factor in factor analysis, indicating that it is possible to simultaneously hold the position of both caring for nature, and for people. However, if the students were displaying a generalised altruism, then the quantitative results might have been expected to show similar scores for both the ecocentrism and anthropocentrism scales, contrary to the findings.

On the other hand, the discrepancy in the findings may reflect complexities within environmental philosophy that cannot be explained by the simple typology of ecocentric and anthropocentric value systems [23] p.9. Amérigo et al. [38] for example, testing the validity of the EAATE scale, proposed a three-dimensional structure to environmental concern: an anthropocentric dimension, a biospheric dimension, and an egobiocentric dimension (the self within nature). Their model is based on the observation that within the original ecocentrism subscale of the EAATE, there are several items that are concerned with the physical or psychological benefits to the individual of interacting with nature, which represents the concept of egobiocentrism: the value placed on the individual within nature, balancing self-interest with respect for the intrinsic value of all living things. In the context of this research, it is possible that some of the high ecocentrism scores found in the quantitative study are explained by responses which represent an egobiocentric, rather than purely ecocentric value position, and that this distinction also clarifies some of the more human-centred comments in the interviews.

A further explanation may be that the high ecocentrism scores reflect ambiguity with the interpretation of some of EAATE items, with students interpreting the meaning of some items differently from how they were intended. This was explored in a study by Kopnina [39], who conducted a qualitative analysis of the EAATE scale involving Business Studies Students, who found some items in the scale to be confusing, ambiguous or open to misinterpretation. Furthermore, within the ecocentrism subscale, there are a lack of statements that test for a genuinely deep green form of ecocentrism, in which human quality of life is traded off against the wellbeing of other species. Deep green ecocentrism represents a more extreme form of ecocentrism, rooted in the deep ecology movement [40], advocating for radical changes in human values, behaviour and economic structure [23] p.67. Thus students with high ecocentrism scores may include not only those with a deep ecological value position, but also those holding less radical, more communalist ecocentric worldviews [23] p.67.

Nevertheless, acknowledging complexities associated with the assessment of environmental value position, in this study levels of apathy towards environmental issues were low, and both the quantitative and qualitative data demonstrated support for ESH, and willingness to engage in ESCP, suggesting that learning approaches focusing on empowerment and agency may be of greatest transformative benefit for environmentally concerned students. For less engaged students, there is also the need for approaches that prompt critical reflection of their environmental worldview.

### Barriers to engaging with sustainable medical behaviour

Despite their environmental concern, and support for ESCP, there appeared to be a disconnection between students’ motivation to behave sustainably and their perceived capacity to do so. They experienced conflict between their conceptualisation of medical professionalism and aspects of sustainable medical practice that were perceived to be at odds with providing optimal patient care. For example, many of the students interviewed described their frustration with the amount of waste that was generated in clinical practice (such as disposable aprons, excessive packaging and single use equipment); however, this was offset by the perception of its necessity for maintaining patient safety and infection control. The findings indicate that, irrespective of how ecocentric the medical students were in their broader environmental worldview, they would prioritise their professional responsibilities over ESCP. Whilst some aspects of waste generation, particularly single use plastic clinical equipment for certain clinical applications, were viewed as non-negotiable for patient safety reasons, other aspects such as pharmaceutical waste, excessive packaging of medical equipment and lack of waste segregation and recycling were felt to be more amenable to change. Although there was a general willingness to address some of these issues, specific solutions (such as sustainable procurement of medical equipment and rational prescribing of medications) were not referenced.

Overall, this sense of frustration and conflict is likely to reflect incomplete integration of ESH into the undergraduate medical curriculum. In particular, it suggests that there is not enough specific guidance about what doctors could and should be doing in terms of promoting environmental sustainability, and, given the importance of socialisation (the internalisation of values and behaviours through interaction with peers and senior clinicians) in medical professional identity formation [41], it also suggests that role modelling of sustainable behaviour is lacking.

### Addressing the barriers to action

One of the main aims of ESH must be to bridge the gap that exists between medical students’ pre-existing environmental concern, and their perceived ability to take positive action in the healthcare setting. Considered within the framework of transformative learning theory, the students appeared to lack both a sense of responsibility and the agency to effect change. Transformative learning approaches for ESH would empower students with the confidence and tools to act, by promoting a greater understanding of doctors’ sustainability roles and responsibilities, encouraging the normalisation of sustainability behaviour, and equipping students with practical skills.

#### Role modelling

Individual and institutional role modelling is a well-recognised component of leadership and change [42], and role modelling of sustainable behaviours has been recognised as an important aspect of leadership in health professional education [43]. The use of role model stories has been studied as an approach to encouraging environmental advocacy in health professionals [44], with promising results in terms of increasing a sense of responsibility for environmental stewardship and willingness to participate in sustainability initiatives. One of the aims of role modelling would be the normalisation of sustainable medical behaviours, such that ESCP is seen as the default way to practice medicine, thereby empowering students with the confidence to undertake specific sustainability actions. Institutional (i.e. university and hospital level) role modelling would involve overt demonstration of sustainable practices such as reduction of waste and single-use plastics (for example: providing access to water fountains rather than selling bottled water, provision of recycling and waste segregation), plant-based menus, use of renewable energy and infrastructure that encourages active transportation [45]. At an individual level, role modelling involves the widespread contribution of engaged faculty and tutors, who can not only communicate the importance of ESCP through case-based learning with examples from clinical practice but also bridge the gap between what is being taught, and what the students are seeing and experiencing on the wards. This might be achieved by the demonstration of specialty specific sustainable practices in the clinical environment, such as inhaler-switching and the adoption of other low-carbon interventions, green and social prescribing, waste segregation, rational use of investigations and practicing realistic medicine [46].

Whilst role modelling can be cultivated within ESH, its impact on students’ sustainability behaviour is likely to be affected by powerful processes such as the hidden curriculum [47], in which learners’ absorb the implicit values, behaviours and norms of an organisation through the culture and observed practices they are exposed to on a daily basis. ESH has the potential to be reinforced or undermined by these experiences, highlighting the importance of addressing not only the formal curriculum, but also medical culture and clinical environments when designing ESH.

#### Specific sustainability skills

Equipping students with specific sustainability skills that they can put into action in their medical practice also represents a transformative approach to ESH. Environmental advocacy in the form of climate and health counselling was identified by the students as a feasible method for doctors to address environmental issues, which might be taught as part of communication skills teaching. This aligns with general recommendations for the inclusion of advocacy as an integral part of ESH [2, 48] and recent empirical work in this area [49–51] investigating the impact of climate sensitive health counselling in both medical education and practice. Climate and health counselling workshops would meet several key criteria in terms of its transformative potential: it represents a clinically relevant, practical skill, that can be taught in an authentic (i.e. real world), interactive way, in small groups via simulation, and which is very amenable to assessment in OSCE examinations, aligning with student preferences for ESH integration. Within ESH frameworks [2, 3], advocacy is a core component of the proposed learning objectives and skills. It is described as occurring at multiple levels: encouraging sustainable practices within clinical settings; influencing organisational/ hospital policy; contributing to public health discourse and climate policy. Activism can also be considered an aspect of sustainable healthcare advocacy and leadership [43]. The questionnaire data indicated 86.1% of students agreed or strongly agreed with the statement “it is appropriate for doctors to engage in environmental activism.” Similarly, in the interviews, students showed support for activism in the context of climate change, but for some students this was tempered with concerns about potential negative impacts on their medical registration and fitness to practice.

#### Transferable skills

In addition to acquiring specific sustainability skills, students can be empowered to enact change through the development of transferable skills. Case studies from the Education for Sustainable Development (ESD) and ESH literature suggest that problem based, action-oriented project work can foster hard and soft skills, which can subsequently be used to address other sustainability challenges. For example, McLean et al. ^52^ describe a team-based planetary health assignment in which small groups of medical students collaborated to develop a creative product that addressed two sustainable development goals, cultivating a range of skills including teamworking, problem solving and use of digital technologies.

#### Worldview shifting

The pedagogical strategies described, such as climate and health communication skills workshops [49–51] and team-based project work [52], address the confidence and competence building aspects of transformative learning. However, of central importance to this theory of learning is a critical assessment of previous understandings of the world, which ultimately leads to a fundamental shift in perspective and actions. Alternative approaches to ESH may also be required to trigger such critical reflection and perspective transformation, particularly in the case of less environmentally engaged students. Interventions that critically examine models of healthcare, resource use, ways of knowing and the environmental impacts of healthcare systems may provide the “disorientating dilemma” described by Mezirow [53] (a profound, unsettling experience that serves to destabilise a person’s existing worldview and forces them to revise their core assumptions and beliefs).

There are robust studies from the ESD and ESH literature that highlight the transformative potential of interventions that challenged students’ worldviews and ways of knowing, through engagement with indigenous knowledge holders [54], via radical eco-pedagogy [55] and through critical reflection[56]. In particular, combining indigenous and Western ways of knowing is thought to be an essential component of addressing current ecological crises [57], which is reflected in growing calls for the inclusion of indigenous perspectives in ESH [2, 58]. Although medical students appear to have high pre-existing levels of environmental concern, learning approaches that focus on third order, epistemic learning, involving shifts in ways of knowing and thinking [13], may have other benefits to medical students beyond shifting their environmental worldview, in terms of instilling a sense of stewardship and responsibility to take pro-environmental action in medicine. A pragmatic approach to ESH would include interventions that address both worldview shifting and bridge the gap between environmental concern and capacity to act through empowerment of students.

### Student preferences for ESH design and integration

The qualitative research took a user-centred approach, giving students a voice in the design of ESH. The findings indicate a strong preference for active learning approaches; in particular, small group learning activities such as discussions, debates and communications skills sessions, delivered in an authentic way by engaged tutors. Clinical relevance was also important, which tended to mean weaving the adverse health outcomes associated with environmental degradation into specific systems teaching, such as cardiovascular or respiratory. Workload and time-pressures were repeatedly cited as barriers to the successful implementation of ESH, the implication being that only content that was summatively assessed, by examination or as part of their academic portfolio, would be prioritised, no matter how interested in a topic they were. Thus, to ensure ESH topics are not overlooked, students suggested integrating them into written or OCSE examinations, portfolio assignments or creating sustainability prizes that would enhance their curriculum vitae. The importance of assessment in providing extrinsic motivation for studying is well recognised in the medical educational literature [2, 59]. The effectiveness of traditional methods of assessment to evaluate ESH has been questioned, due to the inherent need for higher order learning [3], with ESH being more suited to reflective based assessments and group projects [48]. Nevertheless, certain aspects of the ESH curriculum do lend themselves to OCSE based assessment, such as health promotion and environmental advocacy.

## Limitations

### Questionnaire

The EAATE scale [25] was used as it is the only measurement scale that specifically attempts to address the underlying motives for environmental concern (ecocentrism-anthropocentrism), however it is limited by a lack of reported structural validity work [60]. In addition, the sample size of this study is relatively small (108 students) and with a low response rate (8.7%); it is possible that the sample population included more environmentally concerned students than was representative of the whole population, and thus the results may have been influenced by selection bias. This may reflect a broader pattern in which students with a pre-existing interest in sustainability are more likely to engage with such research, further highlighting the importance of integrating sustainability more comprehensively into curricula to foster wider student awareness and participation. Furthermore, the association between ecocentrism and ESCP established by the quantitative study needs to be further tested, ideally in a longitudinal randomized controlled trial, or longitudinal cohort study.

### Interviews

There was potential for bias resulting from the insider researcher status and unequal positioning between the interviewer and students (the interviewer held teaching and leadership roles at the school of medicine in which the research was conducted). A second limitation was the challenge in recruiting students with a broader range of environmental value position; the students who volunteered for the interviews generally held relatively high ecocentrism scores and low apathy scores, resulting in findings that primarily reflect the perspectives of more environmentally concerned students and may not be transferable to less engaged students. The inclusion of students with higher scores on the apathy subscale might have improved the transferability of the findings and represents an important area for future research.

### Generalisability

The findings and implications of this research are potentially generalisable to medical schools in socio-economically similar settings, given the broad similarities in ESH delivery that exist between such medical schools, particularly in relation to longitudinal integration, diverse pedagogical approaches and barriers to implementation [8]. However, the extent to which these findings can be applied to medical students in low and middle-income countries may be limited, due to differences in healthcare infrastructure, funding, health outcomes, relationships with the natural environment and susceptibility to climate change impacts.

## Conclusion

Four recommendations can be made for the design and delivery of transformative ESH in undergraduate medicine, with the aim of empowering students with the confidence and capability to act sustainably. Given the exploratory nature of this research, the impact of these recommended ESH interventions needs to be evaluated, using methods of assessment that focus on higher level transformative outcomes. Four levels of evaluation have been described in relation to educational programs [61]; reaction, learning, behavioural change and impact. In the context of ESH, it is likely that quantifying the environmental impact of a specific learning intervention may be too challenging to be conducted routinely, whereas measuring shifts in environmental worldview and behaviour may represent a more achievable approach, that goes beyond self-reported intentions.

Firstly, it is recommended to include summative assessment as part of the integration of ESH into curricula (via methods such as OSCE stations testing sustainability skills and reflective portfolio assignments). Secondly, design ESH interventions in accordance with students’ learning preferences for interactive, student-centred, experiential approaches, and deliver them in relevant and authentic ways. Thirdly, address the conflict experienced by students in relation to sustainable medical practice and professional identity by empowering them to act using role modelling, hard and soft skills development and project work. Fourthly, incorporate learning approaches which include critical reflection to challenge students’ pre-existing environmental worldviews and epistemologies.

## Supplementary Information

Supplementary Material 1.

## Data Availability

The datasets generated and analysed during this study are available from the corresponding author on reasonable request.

## Abbreviations

BMSc: Bachelor of Medical Sciences
EAATE: Ecocentric and Anthropocentric Attitudes Towards the Environment
ESCP: Ecologically Sustainable Clinical Practice
ESD: Education for Sustainable Development
ESH: Education for Sustainable Healthcare
EVP: Environmental Value Position
OSCE: Objective Structured Clinical Examination
QUAL: Qualitative
QUAN: Quantitative
ScotGEM: Scottish Graduate Entry Medicine
