# Supplementary material for: Designing transformative sustainability education: a mixed methods study exploring the values and perspectives of medical students
Source: BMC Med Educ. 2026 Jul 31;26:1736. doi: 10.1186/s12909-026-09960-8 (PMC13715234; doi:10.1186/s12909-026-09960-8)
Supplement: Supplementary file 1 [file 12909_2026_9960_MOESM1_ESM.docx]

Supplementary data file:

Questionnaire instrument

*EAATE questionnaire:*

Answers are given on a 5-point Likert Scale.

- Strongly disagree
- Disagree
- Neutral
- Agree
- Strongly agree

1. One of the worst things about overpopulation is that many natural areas are getting destroyed for development and agriculture.
2. I can enjoy spending time in natural settings just for the sake of being amongst nature.
3. Environmental threats such as climate change, biodiversity loss and pollution have been exaggerated.
4. The worst thing about deforestation and the loss of the rainforests is that it will restrict the development of new medicines.
5. Sometimes it makes me sad to see forests cleared for agriculture.
6. It seems to me that most environmentalists are pessimistic and somewhat paranoid.
7. I prefer wildlife reserves to zoos.
8. The best thing about camping is that it is a cheap holiday.
9. I do not think the problem of depletion of natural resources is as bad as many people make it out to be.
10. I find it hard to get too concerned about environmental issues.
11. It bothers me that as humans we are running out of our supply of fossil fuels.
12. I need time in nature to be happy.
13. Science and technology will eventually solve our problems with climate change, pollution, overpopulation, and diminishing resources.
14. The thing that concerns me most about deforestation is that there will not be enough timber for future generations.
15. I do not feel that humans are dependent on nature to survive.
16. Sometimes when I am unhappy I find comfort in nature.
17. Most environmental problems will solve themselves given enough time.
18. I don’t care about environmental problems.
19. One of the most important reasons to keep lakes and rivers clean is so that people have a place to enjoy water sports.
20. I am opposed to programs to preserve wilderness, reduce pollution and conserve resources.
21. It makes me sad to see natural environments destroyed.
22. The most important reason for conservation is human survival.
23. One of the best things about recycling is that it saves money.
24. Nature is important because of what it can contribute to the pleasure and welfare of humans.
25. Too much emphasis has been placed on conservation.
26. Nature is valuable for its own sake.
27. We need to preserve resources to maintain a high quality of life.
28. Being out in nature is a great stress reducer for me.
29. One of the most important reasons to conserve is to ensure a continued high standard of living.
30. One of the most important reasons to conserve is to preserve wild areas.
31. Continued land development is a good idea as long as a high quality of life can be preserved.
32. Sometimes animals seem almost human to me.
33. Humans are as much a part of the ecosystem as other animals.

Text in red has been added or modified to reflect current environmental issues. The original scale was published in 1994.

Interpretation:

1. The EAATE scale is composed of three subscales:
   a. Ecocentric
   b. Anthropocentric
   c. Environmental Apathy
2. Answers are given using a 5-point Likert Scale
3. A mean score can be taken for each subscale, ranging from 1 to 5
4. The Ecocentric subscale consists of 12 items (items: 1, 2, 5, 7, 12, 16, 21, 26, 28, 30, 32 and 33). This subscale measures an appreciation for nature, separate from the gains provided for humans. High scores on the Ecocentric subscale indicate a greater appreciation for the environment and positive physiological changes as a result of being in nature
5. The Anthropocentric subscale consists of 12 items (items: 4, 8, 11, 13, 14, 19, 22, 23, 24, 27, 29 and 31). This subscale measures an appreciation for nature, but in respect to the quality of life and survival of humans. High scores on the Anthropocentrism subscale indicate an egoistic concern as in how the environment affects the quality of life for humans
6. The Environmental Apathy subscale consists of nine items (items: 3, 6, 9, 10, 15, 17, 18, 20 and 25). This subscale measures a disbelief in the reality of environmental issues and a lack of interest in these issues. High scores on the Environmental Apathy subscale indicate their strong apathy for environmental issues.

*ESCP questions:*

6 additional questions relating to sustainability in medicine and pro-environmental medical behaviour (devised by lead author):

Answers are given on a 5-point Likert Scale.

- Strongly disagree
- Disagree
- Neutral
- Agree
- Strongly agree

1. *It is important for topics such as climate change, planetary health and sustainability in healthcare to be included in undergraduate medical curricula*
2. *Doctors must ensure that their clinical practice has as low an environmental impact as possible, whilst optimising patient health outcomes*
3. *Doctors must ensure that the department and hospital in which they work are as environmentally sustainable as possible*
4. *Doctors should advise their patients and the public about the health effects of environmental degradation*
5. *Doctors should attempt to influence environmental policy*
6. *It is appropriate for doctors to engage in environmental activism*

Interpretation:

High scores in this section suggest they support education for sustainable healthcare and endorse the role of doctors in practising sustainable medicine and advocating for planetary health

Interview Guide

This interview guide is representative of that used in the semi-structured interviews, however as the interviews progressed and the transcripts were analysed, minor revisions and refinements were continually made, for example, to allow for improvements in the wording of questions, and to address emerging themes.

**Pre-interview introductory process:**

- Personal introductions
- Confirm Participant Information Sheet understood
- Confirm Informed Consent Form understood and signed
- Outline the recording process (i.e. via Teams or Yuja with transcription generation)
- Outline data handling
  - Recorded data stored on One Drive
  - Once accurate transcript generated, recording will be deleted and transcript stored on One Drive
  - Offer to share transcription if desired
- Outline process
  - Purpose of the research
  - No right or wrong answers, their opinions are key
  - Approximately 30mins duration
  - Certificate sent via email

**Part 1: Attitudes towards environmental issues and sustainable healthcare education:**

1. What, if anything, do you know about environmental issues such as climate change, biodiversity loss and pollution?
   a. Prompt: why do you say this?
   b. Prompt: does this relate more generally, or to medicine specifically?
2. What, in your opinion, should individuals and society be doing to address these issues?
3. What, if anything, do you know about the inclusion of topics such as planetary health and sustainable healthcare into medical curricula, generally or at the University of Dundee?
   a. Prompt: can you expand/ tell me why?
4. (Are these topics talked about in clinical practice in your experience?
   a. Prompt: can you give me an example? Where? How?)
5. Do you think these topics should be included? / Why do you think these topics are starting to be taught?
6. What do you consider to be the role of doctors in protecting the natural environment?
   a. What about other healthcare professionals? Who specifically?
7. What is your attitude towards the following activities in medicine:
   a. Environmentally sustainable clinical practice?
   b. Environmental advocacy?
   c. Activism?
      i. Prompt: Such as Doctors for Extinction Rebellion
8. How likely are you to engage in these as a doctor?
   a. Prompt: Can you specify which and why?
9. In your opinion, should environmentalism be part of a doctor’s professional identity?
   a. Prompt: can you expand? / tell me why.
10. What do you know about the concepts of ecocentrism and anthropocentrism?
    a. Prompt: where do you think you sit on the spectrum of environmentalism?
    b. Prompt: do you think this has any relevance to how we should teach PH and SH?

**Part 2: Designing educational approaches for transformative change**

1. What sort of learning methods do you find most impactful and why?
   a. Prompt: can you give me any examples?
   b. Lectures?
   c. Workshops?
   d. Small group discussions?
   e. Projects?
2. How do you think planetary health and sustainable healthcare should be taught in medical school?
   a. Prompt: in what ways?
   b. Prompt: what topics should be taught?
   c. Prompt: what should be the aims/ point of teaching these topics?
3. In your opinion, are three any sorts of creative approaches to learning that might assist with developing impactful solutions to sustainable healthcare education?
4. In your opinion, what sort of educational approaches might help promote a shift in mindset and create transformative changes in medical behaviour and culture?
   a. Prompt: can you explain why?
5. Have you anything else to add, which I have not covered today?

Additional Quantitative Data

Subscale reliabilities

| **Subscale** | **Cronbach’s Alpha** | **Number of items** |
| --- | --- | --- |
| Ecocentrism | .72 | 12 |
| Anthropocentrism | .71 | 12 |
| Apathy | .81 | 9 |
| ESCP | .74 | 6 |

Descriptive Statistics

*EAATE subscale percentage scores split by demographic variables:*

| **Demo-graphic** | **Subgroup (N)** | **Ecocentrism %** | | **Anthropocentrism %** | | **Apathy %** | |
| --- | --- | --- | --- | --- | --- | --- | --- |
|  |  | **Mean (SD)** | **Median (IQR)** | **Mean (SD)** | **Median (IQR)** | **Mean (SD)** | **Median (IQR)** |
| **Gender** | Female (73) | 86.05 (7.58) | 86.67 (11.67) | 53.65 (8.51) | 53.33 (11.67) | 28.49 (8.07) | 26.67 (8.89) |
|  | Male (31) | 84.09 (10.69) | 85.00 (13.33) | 57.53 (11.62) | 58.33 (15.00) | 31.25 (10.53) | 28.89 (13.33) |
|  | Other (2) | 91.67 (7.07) | 91.67 (-) | 43.33 (18.86) | 43.33 (-) | 23.33 (4.71) | 23.33 (-) |
|  | PNTS (2) | 85.00 (0) | 85.00 (0) | 56.67 (4.71) | 56.67 (-) | 22.22 (0) | 22.22 (0) |
| **Ethnicity** | WC (86) | 87.17 (7.42) | 88.33 (8.33) | 54.15 (9.58) | 53.33 (11.67) | 27.42 (8.14) | 24.44 (6.67) |
|  | Asian (14) | 79.52 (10.30) | 80.83 (10.83) | 58.45 (10.73) | 58.33 (11.67) | 37.30 (8.90) | 36.67 (8.33) |
|  | BACBB (3) | 75.00 (4.41) | 73.33 (-) | 48.33 (2.89) | 46.67 (-) | 26.67 (2.22) | 26.67 (-) |
|  | Arab (1) | 66.67 (-) | 66.67 (-) | 61.67 (-) | 61.67 (-) | 46.67 (-) | 46.67 (-) |
|  | MM (3) | 88.33 (7.64) | 90.00 (-) | 60.56 (6.94) | 58.33 (-) | 33.33 (7.70) | 28.89 (-) |
|  | Other (1) | 75.00 (-) | 75.00 (-) | 36.67 (-) | 36.67 (-) | 33.33 (-) | 33.33 (-) |
| **Year of study** | 1 (27) | 84.32 (9.63) | 85.00 (10.00) | 57.72 (9.96) | 56.67 (11.67) | 32.10 (10.68) | 31.11 (17.78) |
|  | 2 (20) | 86.25 (5.48) | 86.67 (7.50) | 49.33 (8.84) | 48.33 (14.58) | 25.56 (6.01) | 24.44 (6.11) |
|  | 3 (15) | 86.00 (6.45) | 86.67 (8.33) | 57.89 (8.13) | 58.33 (8.33) | 27.11 (7.14) | 24.44 (6.67) |
|  | BMSc (12) | 90.42 (8.41) | 93.33 (8.75) | 53.89 (9.14) | 52.50 (12.92) | 26.11 (5.85) | 24.44 (10.00) |
|  | 4 (8) | 81.04 (8.73) | 81.67 (14.17) | 53.75 (12.27) | 49.17 (17.08) | 28.06 (7.51) | 26.67 (7.78) |
|  | 5 (15) | 84.00 (10.48) | 86.67 (15.00) | 56.44 (9.21) | 56.67 (8.33) | 31.56 (10.70) | 26.67 (17.78) |
|  | ScotGEM3 (4) | 86.25 (12.05) | 86.67 (21.25) | 48.75 (10.31) | 50.00 (18.75) | 29.44 (11.09) | 26.67 (20.56) |
|  | ScotGEM4 (7) | 87.38 (7.75) | 90.00 (11.67) | 52.62 (9.62) | 55.00 (8.33 | 32.38 (8.40) | 28.89 (13.33) |

*PNTS = prefer not to say; WC = White/ Caucasian; BACBB = Black/ African/ Caribbean/ Black British; MM = Mixed/ Multiple*

*BMSc = Bachelor of Medical Sciences; ScotGEM = Scottish Graduate Entry Medicine; SD = Standard Deviation; IQR = Interquartile Range*

*ESCP percentage scores split by demographic variables:*

| **Demographic** | **Subgroup** | **N** | **ESCP %** |  |
| --- | --- | --- | --- | --- |
|  |  |  | **Mean (SD)** | **Median (IQR)** |
| **Gender** | Female | 73 | 84.47 (10.99) | 83.33 (16.67) |
|  | Male | 31 | 83.33 (11.71) | 83.33 (16.67) |
|  | Other | 2 | 100 (0) | 100 (0) |
|  | PNTS | 2 | 85.00 (11.79) | 85.00 (-) |
| **Ethnicity** | WC | 86 | 85.74 (10.81) | 86.67 (16.67) |
|  | Asian | 14 | 78.81 (11.95) | 76.67 (15.00) |
|  | BACBB | 3 | 78.89 (7.70) | 83.33 (-) |
|  | Arab | 1 | 60.00 (-) | 60.00 (-) |
|  | MM | 3 | 88.89 (10.18) | 86.67 (-) |
|  | Other | 1 | 80.00 (-) | 80.00 (-) |
| **Year of study** | 1 | 27 | 79.50 (12.25) | 80.00 (16.67) |
|  | 2 | 20 | 87.33 (10.90) | 86.67 (19.17) |
|  | 3 | 15 | 84.44 (10.89) | 83.33 (20.00) |
|  | BMSc | 12 | 90.28 (10.96) | 93.33 (16.67) |
|  | 4 | 8 | 80.00 (9.09) | 80.00 (15.00) |
|  | 5 | 15 | 85.33 (9.41) | 83.33 (16.67) |
|  | ScotGEM3 | 4 | 86.67 (15.15) | 90.00 (28.33) |
|  | ScotGEM4 | 7 | 87.14 (7.56) | 86.67 (10.00) |

*PNTS = prefer not to say; WC = White/ Caucasian; BACBB = Black/ African/ Caribbean/ Black British; MM = Mixed/ Multiple*

*BMSc = Bachelor of Medical Sciences; ScotGEM = Scottish Graduate Entry Medicine*

Inferential Statistics

*Statistical analysis of differences in ESCP percentage score between demographic subgroups:*

|  |  |  | **ESCP %** | **Mann-Whitney** | **Kruskal-Wallis** |
| --- | --- | --- | --- | --- | --- |
| **Demographics** | **Subgroup** | **N** | Median (IQR) | U statistic, two-tailed p value | P value*** from overall test for a difference |
| Ethnicity | White/ Caucasian | 86 | 86.67 (16.67) | 296, p=0.029 | 0.054 |
|  | Asian | 14 | 76.67 (15.00) |  |  |
|  | Rest * | 8 | 81.67 (13.33) |  |  |
| Gender | Female | 73 | 83.33 (16.67) | 1055, p=0.586 | 0.314 |
|  | Male | 31 | 83.33 (16.67) |  |  |
|  | Rest ** | 4 | 96.67 (19.17) |  |  |
| Year of study | Years 1-3 | 62 | 83.33 (16.67) | - | 0.158 |
|  | BMSc | 12 | 93.33 (16.67) |  |  |
|  | Years 4-5/ ScotGEM | 34 | 83.33 (16.67) |  |  |

IQR = Interquartile Range; *BMSc = Bachelor of Medical Sciences; ScotGEM = Scottish Graduate Entry Medicine*

* Less than 10 students in the Rest category so this group not included in Mann-Whitney test

** Less than 10 students in the Rest category so this group not included in Mann-Whitney test

*** p value from two-tailed test for a difference in the mean ranks of at least one of the pairs of groups

*Spearman's Rank Correlations of subscale scores and continuous demographic variables with ESCP score:*

| Independent variable | Correlation coefficient (r) | p-value (2 tailed) |
| --- | --- | --- |
| Ecocentrism | .482** | < 0.001 |
| Anthropocentrism | -.196* | .042 |
| Apathy | -.540** | < 0.001 |
| Age | .219* | .023 |

**. Correlation is significant at the 0.01 level (2-tailed).

*. Correlation is significant at the 0.05 level (2-tailed).

ESCP: Ecologically Sustainable Clinical Practice

Univariable regression outputs

|  |  | Unadjusted |  |  |  | Adjusted^ψ^ |  |  |  |
| --- | --- | --- | --- | --- | --- | --- | --- | --- | --- |
|  |  | Coefficient | Standard error | Z value | P value | Coefficient | Standard error | Z value | P value |
| Ethnicity | Intercept | 86.19 | 1.25 | 68.70 | <0.0001*** | 86.93 | 1.45 | 59.99 | <0.0001*** |
|  | WC | Ref | Ref | Ref | Ref | Ref | Ref | Ref | Ref |
|  | Asian | -8.05 | 3.35 | -2.40 | 0.016* | -5.77 | 3.38 | -1.71 | 0.087 |
|  | Other | -5.54 | 4.30 | -1.29 | 0.198 | -4.33 | 4.22 | -1.03 | 0.305 |
|  | (Age centred) | - | - | - | - | 1.11 | 0.52 | 2.12 | 0.034* |
|  | (Age centred) ^2^ | - | - | - | - | -0.17 | 0.10 | -1.62 | 0.105 |
| Year of Study | Intercept | 83.63 | 1.49 | 56.26 | <0.0001*** | 87.82 | 2.12 | 41.41 | <0.0001*** |
|  | 1-3 | Ref | Ref | Ref | Ref | Ref | Ref |  | Ref |
|  | BMSc | 7.23 | 3.69 | 1.96 | 0.0503 | 3.27 | 3.84 | 0.85 | 0.395 |
|  | 4-5/ ScotGEM | 0.93 | 2.50 | 0.37 | 0.710 | -5.77 | 3.32 | -1.74 | 0.082 |
|  | (Age centred) | - | - | - | - | 2.03 | 0.68 | 2.99 | 0.003** |
|  | (Age centred) ^2^ | - | - | - | - | -0.24 | 0.11 | -2.15 | 0.032* |

^ψ^ adjusted for age and age2. Significance: * < 0.05; **<0.01; ***<0.001

There was no evidence for an association of ESCP with ethnicity after adjusting for age (F = 1.76, p = 0.177), however this may reflect the small sample size. Similarly, there was little evidence for a difference in ESCP score by year of study in an age-adjusted model (F = 2.85, p = 0.063).

Qualitative study

*Participant demographics:*

|  | **Year of study** | **Age** | **Ethnicity** | **Gender** | **Eco score (/60)** | **Anthro score (/60)** | **Apathy score (/45)** | **ECSP score (/25)** |
| --- | --- | --- | --- | --- | --- | --- | --- | --- |
| Participant 1 | Year 1 | 18 | WC | M | 53 | 48 | 11 | 25 |
| Participant 2 | Year 2 | 20 | WC | F | 54 | 37 | 12 | 24 |
| Participant 3 | Year 1 | 18 | WC | F | 57 | 41 | 11 | 25 |
| Participant 4 | BMSc | 21 | WC | M | 57 | 26 | 10 | 29 |
| Participant 5 | Year 2 | 19 | WC | M | 51 | 26 | 9 | 30 |
| Participant 6 | Year 3 | 26 | WC | F | 49 | 32 | 13 | 24 |
| Participant 7 | Year 5 | 23 | WC | M | 47 | 37 | 21 | 25 |
| Participant 8 | Year 2 | 19 | WC | F | 52 | 35 | 9 | 30 |
| Participant 9 | Year 5 | 22 | WC | F | 48 | 33 | 19 | 24 |
| Participant 10 | Year 4 | 30 | Asian | M | 44 | 39 | 18 | 22 |

*Eco = Ecocentrism; Anthro = Anthropocentrism; ESCP = Ecologically Sustainable Clinical Practice; M = Male; F = Female; WC = White Caucasian*

*Summary of major themes and sub-themes:*

| **Major themes and sub-themes** | **Clusters** | **Relationship with research aims** |
| --- | --- | --- |
| **Human-centredness**  **Emotional turmoil**   - Frustration - Eco-anxiety - Guilt |  | Environmental value position and perspectives on environmental issues |
| **Ecological health consciousness**   - Support for ESH   **Conflict and cognitive dissonance**   - The patient comes first - Frustration at the environmental impact of healthcare - The need for top-down leadership   **Support for advocacy**   - Patient consultations - Activism | The impact of waste generation | Perspectives on ESH and ESCP |
| **Preference for active learning**   - Small group sessions - Projects and challenges   **Necessity of relevance**   - Academic relevance - Clinical relevance   **Importance of authenticity**   - Tutor engagement - Role modelling | Communications skills sessions  Interactive lectures  Online modules  Assessment  Rewards and prizes  Outside speakers | Preferences for the design of ESH |

*ESH = Education for Sustainable Healthcare; ESCP = Ecologically Sustainable Clinical Practice; EAATE = Ecocentric and Anthropocentric Attitudes Towards the Environment; QUAL = Qualitative; QUAN = Quantitative*

*Summary of integrated findings*

| **Key themes** | **QUANTITATIVE findings** | **QUALITATIVE findings** | **Integration/ recommendation** |
| --- | --- | --- | --- |
| Environmental Value Position | High ecocentrism, moderate anthropocentrism, low apathy scores. Positive correlation between ecocentrism and support for ESCP. | More complex basis of environmental concern. Negative emotional responses to environmental issues. | High levels of environmental concern but underlying value basis less clear, particularly in the context of medicine due to conflict with professional identity. Association between ecocentrism and ESCP requires further investigation. |
| Conflict | High ESCP scores | Support for ESCP, including environmental advocacy, particularly at patient level.  Conflict regarding certain aspects of sustainable medical practice. Patient safety prioritized regardless of ecocentrism score. | Need for greater empowerment and agency through role modelling and skills development (specific sustainability skills and transferable skills). Use of critical reflection to foster sense of stewardship and empowerment. |
| Summative assessment | Not explored in QUANTITATIVE study. | Importance of summative assessment in determining prioritization of topics in medicine. | Integration of sustainability topics into online examinations, OSCEs, project work, and reflective portfolio  assignments to maximise engagement. |
| Learning preferences | Not explored in QUANTITATIVE study. | Support for ESH. Preference for active, small group, experiential learning, delivered in an authentic and clinically relevant way. | Design and deliver ESH teaching according to student preferences; evaluate effectiveness of ESH interventions focusing on higher level transformative outcomes. |

*ESCP = Ecologically Sustainable Clinical Practice; ESH = Education for Sustainable Healthcare; OSCE = Objective Structured Clinical Examination*
